# Three-dimensional culture models mimic colon cancer heterogeneity induced by different microenvironments

**DOI:** 10.1038/s41598-020-60145-9

**Published:** 2020-02-21

**Authors:** Shigeto Kawai, Masaki Yamazaki, Keita Shibuya, Masaya Yamazaki, Etsuko Fujii, Kiyotaka Nakano, Masami Suzuki

**Affiliations:** 1 Department for Research Division 1, Forerunner Pharma Research Co., Ltd., 4–6–1, Komaba, Meguro-ku, Tokyo 153–8904 Japan; 2 Research Division, Chugai Pharmaceutical Co., Ltd., 1–135, Komakado, Gotemba, Shizuoka 412–8513 Japan

**Keywords:** Cancer microenvironment, Cancer stem cells

## Abstract

Colorectal cancer demonstrates intra-tumour heterogeneity formed by a hierarchical structure comprised of cancer stem cells (CSCs) and their differentiated progenies. The mechanism by which CSCs are maintained and differentiated needs to be further elucidated, and there is evidence that the tumour microenvironment governs cancer stemness. Using PLR123, a colon cancer cell line with CSC properties, we determined the culture conditions necessary to establish a pair of three-dimensional (3D) culture models grown in Matrigel, designated stemCO and diffCO. The conditions were determined by comparing the phenotypes in the models with PLR123 mouse xenografts colonising lung and liver. StemCO resembled LGR5-positive undifferentiated tumours in the lung, and diffCO had lumen structures composed of polarised cells that were similar to the ductal structures found in differentiated tumours in the liver. In a case using the models for biomedical research, treatment with JAG-1 peptide or a γ-secretase inhibitor modified the Notch signaling and induced changes indicating that the signal participates in lumen formation in the models. Our results demonstrate that culture conditions affect the stemness of 3D culture models generated from CSCs and show that comparing models with different phenotypes is useful for studying how the tumour environment regulates cancer.

## Introduction

Colorectal cancers, especially differentiated types that form ductal structures, are composed of a heterogeneous population of undifferentiated cancer stem cells (CSCs) and differentiated cells, constituting a hierarchical structure. The tumour microenvironment has an important effect on CSC maintenance and differentiation, which in turn affects the hierarchy of a cancer. CSCs reside in a tumour-host interface (the so-called stem-niche) in colorectal cancer, and their stemness is speculated to be maintained by host-derived factor(s)^1–3^. Therefore, reproducing a precise tumour microenvironment is crucial for modeling the cancer hierarchy *in vitro*.

Three-dimensional (3D) cultures are frequently used for studying biology of epithelial tissues since they can reproduce the 3D organisation and function of cells within tissues^4^. They have been used to elucidate the contribution of microenvironmental factors to normal and disease processes and to advance therapeutic approaches^4–6^. One of the 3D culture techniques is the organoid culture, with which normal intestinal stem cells generate organoids with crypt-villus structures^4^. Organoid cultures are also used to culture cancer cells from patient tumour samples since they can recapitulate the structural and functional heterogeneity of the original tumour^7^. If the phenotypes of 3D cultures derived from colorectal CSCs can be controlled by adjusting culture conditions, then the cultures would be useful for analysing the relationship between CSC maintenance and differentiation induced by the tumour environment. To this end, the appropriate conditions should be tested using well-characterised CSCs.

We established a colon cancer cell line with CSC properties, PLR123, from a patient-derived xenograft model in immunodeficient NOG mice^8^. PLR123 cells are maintained by monolayer culture in a serum-free medium containing EGF and FGF2, which we designated as the stem cell medium (SCM). The cells express stem cell markers, including Leucine-rich repeat-containing G-protein coupled receptor 5 (LGR5), and either self-renew by symmetric cell division or differentiate by asymmetric cell division^8^. Furthermore, they are highly tumourigenic in NOG mice and generate tumours with a differentiated ductal structure which closely resembles the structure of the original patient tumour. Since the ability of PLR123 cells to self-renew or differentiate is well-validated, the cell line was considered ideal material for generating 3D culture models possessing different phenotypes by modifying culture conditions.

Here, we generated a pair of 3D culture models using PLR123 cells. One model retained stemness during growth (stem-cancer organoid or stemCO); the other model had lumen structures consisting of polarised differentiated cells (diff-cancer organoid or diffCO). By comparing these models, we could analyse the phenotypic changes of CSCs corresponding to their environment.

## Results

### PLR123 cells formed tumours with distinct features in mouse liver and lung

**Figure 1:**
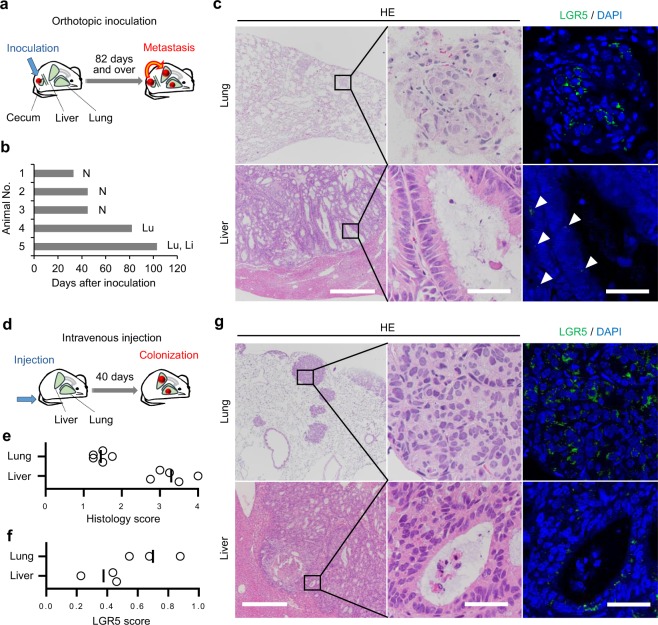
PLR123 cells generate phenotypically different tumours in mouse lung and liver. (**a**) Schematic of the orthotopic inoculation experiment. PLR123 cells were inoculated to cecum of NOG mice (5 mice). (**b**) Distant metastases to lung and liver were observed in mice which survived 82 days and over after inoculation. N: no metastasis, Lu: metastasis to lung, Li: metastasis to liver. (**c**) Histology and LGR5 immunostaining of the metastases. Arrowheads indicate LGR5 positive tumours in liver. (**d**) Schematic of the intravenous injection experiment. PLR123 cells were injected via the tail vein of NOG mice (5 mice). Tumour colonization in lung and liver was confirmed in all mice 40 days after injection. (**e**) A histology score was calculated for each mouse, as described in Methods. (**f**) An LGR5 score was calculated for 3 mice, as described in Methods. (**g**) Histology and LGR5 immunostaining of the tumours. Scale bars = 500 μm (left figures) or 40 μm (center and right figures) (**c,g**).

Using PLR123 cells as the material for 3D culture models, we confirmed the potential of the cells to change phenotype according to their environment. Although PLR123 cells inoculated subcutaneously into NOG mice form tumours with differentiated structures^8^, the histological features of tumours generated in environments other than the subcutis was unknown. Therefore, we evaluated PLR123 cells in a metastasis model. Since inoculating cancer cells at the orthotopic site is a common approach for generating distant metastasis^9,10^, we first transplanted PLR123 cells to the cecum of NOG mice (Fig. 1a). As a result, tumours in the lung and liver were confirmed at approximately 100 days after inoculation in 2 out of 5 mice tested (Fig. 1b). The other 3 mice were sacrificed relatively early because of deterioration of general conditions due to obstruction of the intestine. Histopathologically, the tumours in the lung predominantly consisted of small nests of cuboidal cells with centrally located nuclei which are features of an unclearly polarised, undifferentiated phenotype (Fig. 1c). In contrast, tumours in the liver consisted of large nests with ductal structures, formed by columnar tumour cells with basal location of nuclei which indicates apicobasal polarisation and differentiation (Fig. 1c). By immunofluorescence staining of LGR5, the tumour cells in the lung tended to have large patchy staining in the cytoplasm compared to small dotty staining in the liver (Fig. 1c). This staining pattern was thought to indicate higher expression levels of LGR5 in the lung tumours. As the incidence of metastasis with orthotopic inoculation was low, and because of the prolonged period needed for its development, it was difficult to judge whether these histological features were due to environmental differences or genetic alterations in the metastasizing cells. So we next attempted to explore a model of intravenous inoculation via the tail vein (Fig. 1d). With intravenous inoculation, PLR123 cells homed in on the lung and liver in all mice within a shorter period of time compared to orthotopic inoculation. By comparing the histology scores, we found that the tumours in the liver had a morphologically differentiated phenotype compared to the tumours in the lung (Fig. 1e,g). Furthermore, scoring of LGR5-positive cells revealed that there was an enrichment of LGR5-positive cells in the lung tumours (Fig. 1f,g). Thus we confirmed the augmented tumour differentiation in liver compared to lung. From these experiments, it was demonstrated that PLR123 cells in different environments form phenotypically different tumours. Therefore, PLR123 cells can feasibly be used to generate phenotypically different 3D culture models by modifying the culture conditions.

### Generation of culture conditions for 2 types of 3D culture models

**Figure 2:**
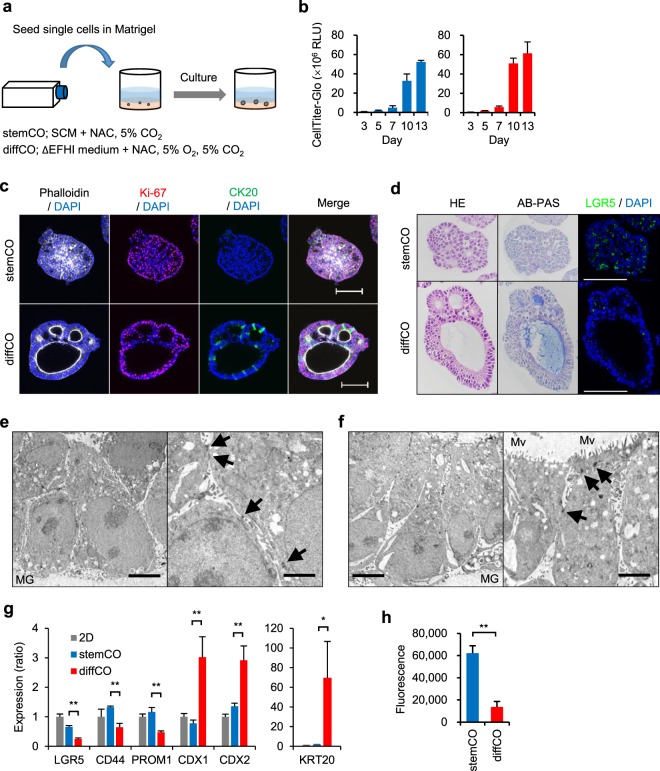
Generation and characterization of PLR123 stemCO and diffCO. (**a**) Schematic of experimental conditions for 3D culture models. (**b**) Time course of stemCO (blue) and diffCO growth (red) analysed by CellTiter-Glo. Data represent mean + s.d. (n = 6). (**c**) Whole mount immunostaining of stemCO and diffCO on Day 10 with phalloidin (white), DAPI (blue), anti-Ki-67 antibody (red), and anti-CK20 antibody (green). Scale bar = 100 μm. (**d**) Day 10 stemCO and diffCO sections were stained with HE, AB-PAS, anti-LGR5 antibody (green), or DAPI (blue). Scale bar = 100 μm. (**e,f**) Transmission electron microscopy of stemCO (**e**) and diffCO (**f**) on Day 10. Arrows, desmosomes; MG, Matrigel; Mv, microvilli. Scale bar = 5 μm (left) or 2 μm (right). (**g**) Expression of CSC (*LGR5*, *CD44*, *PROM1*) and differentiation markers (*CDX1*, *CDX2*, *KRT20*) in Day 10. StemCO and diffCO was analysed by quantitative RT-PCR. The relative expression ratio was calculated in relation to a 2D control. Data represent mean + s.d. (n = 3). *p < 0.05; **p < 0.01, Student *t* test. (**h**) Colony formation assay of single cells derived from Day 10 stemCO or diffCO. Cells were seeded at 10,000 cells per well in 96-well plates containing soft agar and cultured for 7 days. Cell growth was quantified using CyQuant dye. Data represent mean + s.d. (n = 4). **p < 0.01, Student *t* test.

We attempted to generate PLR123 3D culture models replicating the histological differences in PLR123 tumours in lung and liver. In the body, partial oxygen pressure (pO_2_) differs widely among organs and tissues. In certain areas of the lung, pO_2_ is close to atmospheric pO_2_ (around 150 mmHg)^11^, but in the liver, it is lower (30–65 mmHg)^12^. Consequently, metastases of colorectal cancer to the liver tend to be hypoxic^13^. Therefore, we chose atmospheric pO_2_, which is a typical condition in cell cultures, and physiological 5% pO_2_ (36 mmHg)^14^. We also tested the antioxidant N-acetylcysteine (NAC), which is frequently used in organoid cultures of colon cancer^15^. Furthermore, we focused on growth factors EGF and FGF2, which are pivotal in sustaining the stemness of colorectal CSCs^16^. Results showed that addition of NAC to the SCM accelerated 3D culture growth and removal of EGF or FGF2 or the use of culture with 5% pO_2_ atmosphere promoted both growth and lumen formation. Thus 2 culture conditions were established; the culture condition of SCM supplemented with NAC and atmospheric pO_2_ generated 3D cultures with minimal lumen formation, and the culture condition of low growth factor medium (ΔEFHI, in which EGF, FGF2, heparin, and insulin were omitted from SCM) supplemented with NAC and 5% pO_2_ generated 3D cultures with marked lumen formation. We designated these models as stemCO and diffCO, respectively (Fig. 2a).

### Characteristics of stemCO and diffCO

Because stemCO and diffCO increased significantly in size from Day 7 to Day 10, Day 10 cultures were analyzed to characterise them (Fig. 2b). Both whole mount immunostaining and histopathological analysis demonstrated spherical solid cell clusters and lumen structures in stemCO and diffCO, respectively (Fig. 2c,d). CSC marker LGR5 was highly expressed in stemCO and differentiation marker CK20 in diffCO (Fig. 2c,d). Proliferation marker Ki-67 was detected in both cultures (Fig. 2c). The labeling index for Ki-67 in immunohistochemical slides was 57.4% and 78.4% for stemCO and diffCO, respectively. Histopathologically, stemCO consisted of round-shaped cells with unclear lumen formation (Fig. 2d). In contrast, diffCO consisted of columnar cells that formed clear lumens. There were mucins within the lumens revealed by positive staining with Alcian blue, indicating the presence of secretory lineage cells. When the cultures were observed by transmission electron microscopy, both structures formed desmosomes, indicating that they retained epithelial features (Fig. 2e,f). In addition, microvilli were observed on the luminal side of the cells in diffCO as a signature of enterocyte-like cells (Fig. 2f). Thus, stemCO showed an undifferentiated morphology compared to the more differentiated diffCO. Expression of CSC markers *LGR5*, *CD44*, and *PROM1* in stemCO and of differentiation markers *KRT20*, *CDX1*, and *CDX2* in diffCO was further confirmed by quantitative real-time PCR (RT-PCR) (Fig. 2g). Finally, a colony formation assay with single cells prepared from each type of culture indicated a significant loss of colony-forming activity in the diffCO cells (Fig. 2h). We speculated that there was lower maintenance of stemness in diffCO, however, both stemCO and diffCO could be maintained for a longer period (42 days) by serial passaging the culture by fragmentation (Supplemental Fig. S1). After this longer culture, LGR5-positive cells were still retained in both stemCO and diffCO, indicating that CSCs were maintained autonomously in diffCO as well as in stemCO (Supplemental Fig. S1). We further asked if diffCO regains stemness when the culture condition was switched to stemCO condition and found the number of LGR5-positive cells were lower than that of stemCO (Supplemental Fig. S2). We speculated that only diffCO cells maintaining sufficient stemness generate LGR5-positive progeny after switching to stemCO condition, and more differentiated cells do not. From these results, we concluded that stemCO mimics the 3D proliferation of CSCs, while diffCO resembles the differentiation and organization of ductal structures characteristic of differentiated colorectal cancer.

### Temporal process of lumen formation in diffCO

**Figure 3:**
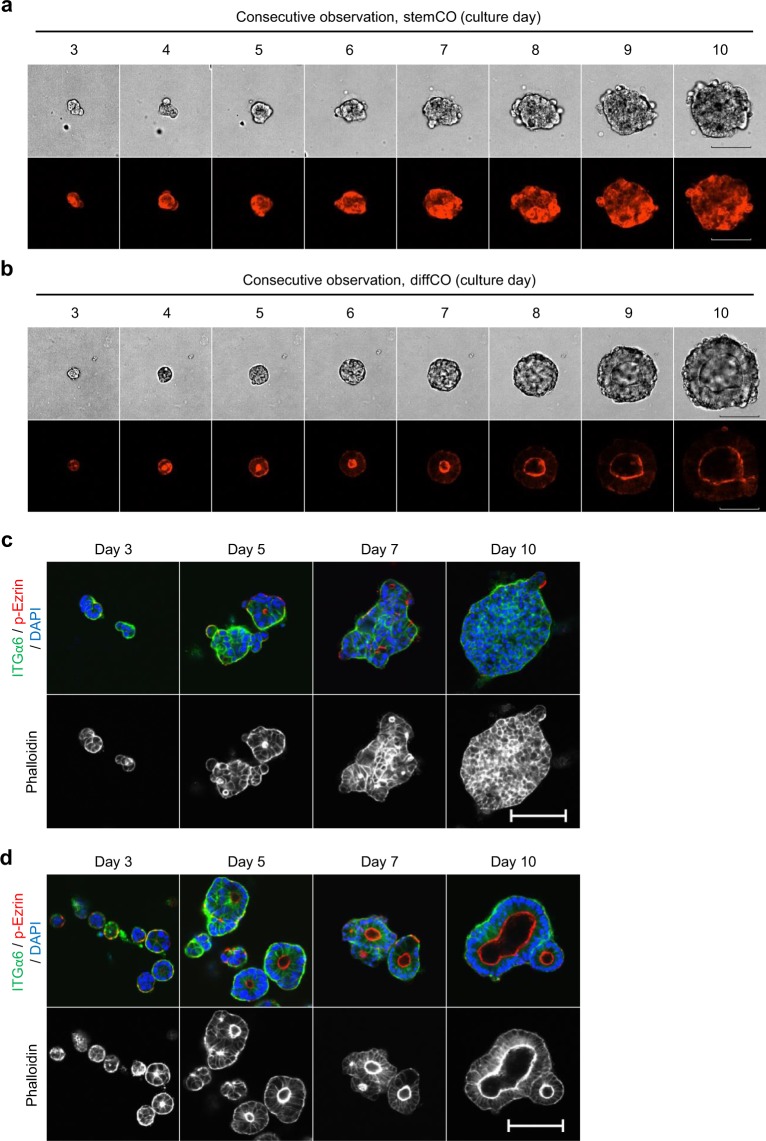
Time course observation of lumen formation in PLR123 stemCO and diffCO. (**a,b**) Bright-field (upper) or confocal fluorescence live-imaging of F-actin (lower) in stemCO (**a)** or diffCO (**b**) in PLR123 cells expressing LifeAct-RFP. (**c,d**) Whole mount immunostaining of Integrin α6 (ITGα6, green), phospho-Ezrin (p-Ezrin, red), phalloidin (white), and DAPI (blue) in stemCO (**c**) and diffCO (**d**). Scale bars = 100 μm (**a–d**).

Confocal time-lapse imaging was used to assess how these lumen structures formed over time in PLR123 cells transfected with LifeAct-RFP, which can visualise F-actin in living cells^17^. StemCO grew as cellular aggregates without showing any clear lumen formation as expected (Fig. 3a). In contrast, in diffCO lumens appeared early and the diffCO grew while maintaining the structure (Fig. 3b). Lumens were also evident in the whole mount immunostaining of diffCO from Day 5 confirmed by apical localisation of phospho-Ezrin and F-actin and basal localisation of Integrin α6 (Fig. 3c,d). Thus, the results suggested that PLR123 cells alter their phenotypes swiftly and develop a lumen structure in response to the diffCO culture condition.

### Application of stemCO and diffCO to the analysis of lumen formation

**Figure 4:**
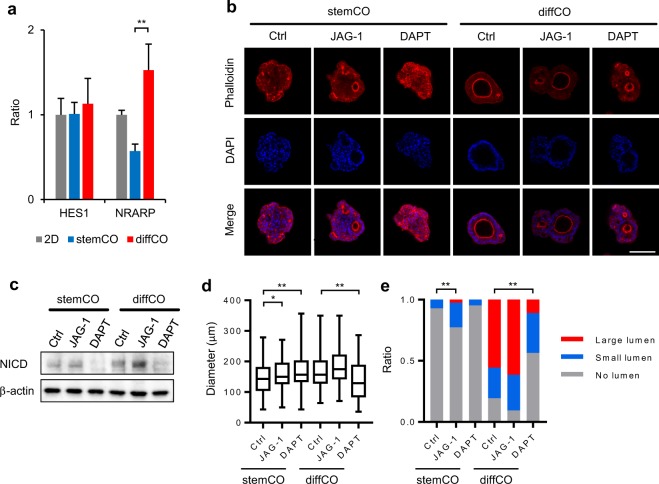
Notch signaling is associated with lumen formation in PLR123 stemCO and diffCO. (**a**) Expression of Notch markers *HES1* and *NRARP* in Day 10 stemCO and diffCO was analysed by quantitative RT-PCR. The relative expression ratio was calculated in relation to a 2D control. Data represent mean + s.d. (n = 3). **p < 0.01, Student *t* test. (**b**) Representative whole mount immunostaining images of phalloidin (red) and DAPI (blue) in stemCO and diffCO treated with DMSO control, 20 μM JAG-1, or 10 μM DAPT on Days 3–10. Scale bar = 100 μm. (**c**) Cropped images of western blot analysis of Notch intracellular domain expression in stemCO and diffCO treated with DMSO control, 20 μM JAG-1, or 10 μM DAPT on Days 3–10. β-actin was used as a control. Full-length blots are presented in Supplementary Fig. S3. NICD: Notch intracellular domain. (**d**) The longitudinal diameter of stemCO and diffCO found in each condition in (**b**) (between 84 and 125 stemCO or diffCO each) was calculated and represented as box-and-whisker plot. *p < 0.05; **P < 0.01, Dunnett’s test compared with DMSO control. (**e**) The longitudinal diameter of the largest lumen in each stemCO and diffCO in (**b**) were measured and the percentages in relation to the size of the corresponding stemCO or diffCO were calculated. Each was classified as having large lumens (≥25%), small lumens (<25%), or no lumens. Data represent the ratio of stemCO and diffCO with large, small, or no lumens in each condition. **p < 0.01, Chi-squared test compared with DMSO control. For the statistical analysis of stemCO, large and small lumens were combined to increase the expected frequency.

To determine whether these 3D culture models could be effectively utilised to analyse the biological properties of PLR123 cells, we compared the responses of the 2 models to external stimuli. We focused on Notch signaling, which is involved in the differentiation of enterocytes in the normal intestine^18^ and is also activated in colorectal CSCs by cellular hypoxic response^19,20^, and anticipated its probable activation in diffCO. Indeed, we detected Notch activation in diffCO, as demonstrated by augmented expression of a Notch-target gene *NRARP* (Fig. 4a). The expression level of another Notch-target gene *HES1* was not changed, but this result was difficult to interpret, because the molecule is also induced by β-catenin, which is often aberrantly activated in colorectal cancer^21^.

We next treated these models with JAG-1 peptide or the γ-secretase inhibitor DAPT to augment or block Notch signaling, respectively, and evaluated their structures (representative images shown in Fig. 4b). Notch signaling was verified by expression of Notch intracellular domain (Fig. 4c). When the longitudinal diameter of stemCO and diffCO were measured as an index for their size, they were slightly affected by JAG-1 peptide and/or DAPT (Fig. 4d). To determine the influence on lumen formation, the longitudinal diameter of the largest lumen in each stemCO and diffCO were measured and the percentages in relation to the size of the corresponding stemCO or diffCO were calculated. Each was classified as having large lumens (≥25%), small lumens (<25%), or no lumens. The number of stemCO with lumens increased significantly with the JAG-1 peptide (Fig. 4e), whereas for diffCO, significant difference was detected with DAPT, presumably resulting from the decrease in the number of diffCO with large-lumens and the increase in those with no-lumens (Fig. 4e). Thus, we confirmed that Notch signaling participates in the formation of lumens in stemCO and diffCO.

### General applicability of stemCO and diffCO culture conditions

**Figure 5:**
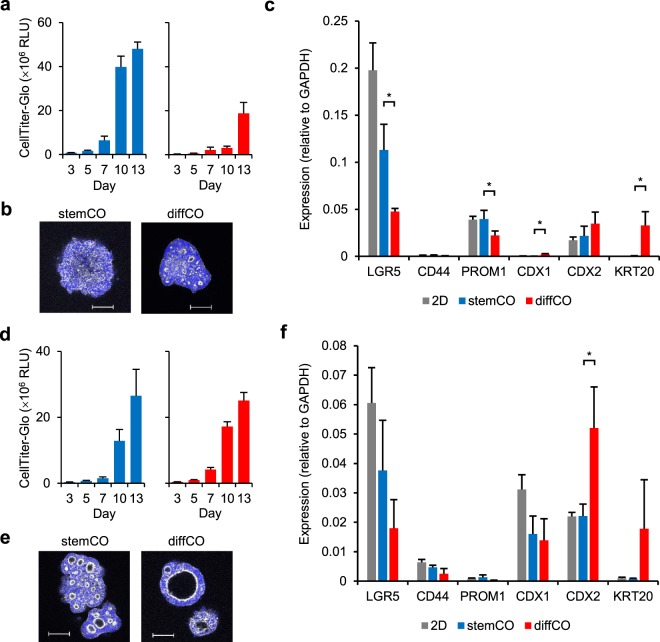
3D cultures of colorectal cancer cell lines LoVo and LS174T. LoVo cells (**a–c**) or LS174T cells (**d–f**) were adapted to SCM and used for 3D cultures. (**a,d**) Growth of the 3D cultures was analysed with CellTiter-Glo. Data represent mean + s.d. (n = 6). (**b,e**) Representative whole mount immunostaining images of phalloidin (white) and DAPI (blue) in cultures on Day 13. Scale bars = 100 μm. (**c,f**) Expression of CSC (*LGR5*, *CD44*, *PROM1*) and differentiation (*CDX1*, *CDX2*, *KRT20*) markers by quantitative RT-PCR analysis. With LoVo cells, cultures on Day 10 and 13 were examined for stemCO and diffCO culture conditions, respectively. With LS174T cells, cultures on Day 13 were examined for both culture conditions. Data represent mean + s.d. (n = 3). *p < 0.05, Student *t* test.

Finally, the 2 culture conditions were applied to commercially available colorectal cancer cell lines LoVo and LS174T, which express LGR5^22,23^. In stemCO culture condition, LoVo cells grew as cell aggregates without forming lumens, whereas diffCO culture condition induced multiple small lumens (Fig. 5a,b). LS174T cells were more prone to forming lumens in both culture conditions (Fig. 5d,e); however, the size of the lumens was larger in diffCO culture condition (Fig. 5e). Furthermore, a decrease in CSC markers *LGR5* and *PROM1* and an increase in differentiation markers *CDX1* and *KRT20* were evident with LoVo cells in the diffCO (Fig. 5c). Differentiation marker *CDX2* was increased with LS174T cells in the diffCO (Fig. 5f). Therefore, although a limited number of cell lines were tested, it was apparent that diffCO culture condition tended to induce differentiation more than stemCO. As a result, these culture conditions are expected to be applicable to cell lines other than PLR123 and to be a useful model to elucidate the biology of colorectal cancers.

## Discussion

It is reported that colorectal cancer exhibits a hierarchical structure influenced by its microenvironment. Because the cancer microenvironment varies, it is important to study which culture conditions would optimally reproduce it. To this end, we developed a pair of 3D culture models for PLR123 cells, which possess the capacity to self-renew and differentiate^8^. StemCO contained cells with high expression of the CSC markers *LGR5*, *CD44*, and *PROM1*, as well as a colony-forming activity, and were thought to be clusters of cells retaining the stem cell properties. On the other hand, diffCO was constituted from more differentiated cells which showed cell polarity and formed lumen structures. When compared with the morphology of the tumours in mice inoculated with PLR123 cells, stemCO resembled LGR5-positive undifferentiated tumours in the lung, and diffCO had similar morphology to differentiated tumours containing ductal structures and a limited number of LGR5-positive cells in the liver. Therefore, by fine-tuning the culture conditions, we have shown that 3D culture models can reproduce the phenotypic differences observed *in vivo*, such as LGR5 positivity or formation of ductal structures, which are presumably governed by their environment.

Culture conditions of the models were determined by focusing on supplementation with growth factors and pO_2_. Growth factors EGF and FGF2 greatly influenced the phenotype of the PLR123 3D culture models, although PLR123 cells carry an activating mutation in *KRAS*^8^. It was reported that the leading tumour edge of colorectal cancer shows high MAPK activity irrespective of *KRAS* mutation status, and that the activity correlates with the expression of CSC markers^24^. Given that stromal cells localised at the tumour-host interface may secrete growth factors such as EGF and FGF2^25,26^, growth factors were considered to constitute a substantial part of the microenvironment that influences the stemness of cancer cells. Therefore the effect of adding growth factors in 3D culture models should be carefully evaluated.

Attenuation of pO_2_ to physiological 5% pO_2_ promoted the growth of 3D culture models. In the case of human embryonic stem cells or mesenchymal stem cells, culture under physiological pO_2_ induces a glycolysis-dominant state^27,28^. When taking into account that colorectal CSCs primarily use mitochondrial OXPHOS rather than glycolysis to generate ATP^29^, management of pO_2_ should also be considered, especially when CSC cultures are used to analyse metabolic regulation.

Having established 2 culture models with different phenotypes, we assumed that comparing the response of the models to external stimuli could aid in analysing the biological properties of PLR123 cells. To prove this concept, we chose Notch signaling, because it is involved in the differentiation of normal intestinal stem cells into enterocytes^18^, which are highly polarised columnar cells constituting ductal architecture of the villi^30^, and may thus be associated with lumen formation. Furthermore, it was reported that Notch signaling is activated in the center of colorectal cancers^31^ in which differentiated tumour cells forming glandular structures can be observed^32^. As expected, Notch signaling was indeed related to lumen formation in PLR123 stemCO and diffCO, an interpretation made possible by comparing the 2 models. We believe this approach is potentially applicable to other models such as patient-derived cancer organoids.

We next validated the culture conditions with other colorectal cancer cell lines, LoVo and LS174T, since optimum culture conditions may vary according to the genetic profiles of the cells^33^. As a result, both cell lines could be cultured in stemCO and diffCO culture conditions, and lumen formation was augmented with the diffCO culture condition. However, the structural difference between the 2 culture conditions was less prominent than it was in PLR123 cells. This might reflect the limited inherent potential of LoVo and LS174T cells to reconstitute hierarchical tumour organization^34,35^, although further analysis is needed.

In cancer biomedical research, patient-derived cancer organoid biobanks are generated and utilised for studies, such as drug screening to aid precision medicine^36–38^. By engrafting these organoids to mice, differentiation and metastases of CSCs are also analysed^3^. An advantage of using xenografts is that the involvement of host-derived factors in the tumour microenvironment contributes to the reproduction of a more clinically relevant condition. On the other hand, *in vitro* models are more advantageous than xenografts in certain processes, such as high-throughput screening, detailed signal pathway analysis, or metabolomic analysis. Therefore, our current findings may support a range of studies with cancer organoids in the future. However a caveat is that colorectal CSCs generally reside in a multicellular organisation while maintaining proper cell-cell interaction within tumour tissues. This property is reproduced in patient-derived organoid cultures but not in 3D cultures generated from homogenous cell lines. Therefore the differences in the culture modalities should be carefully examined.

In conclusion, we developed 2 types of 3D culture models with PLR123 cells possessing CSC properties. The models responded differently to a small molecule inhibitor or ligand, as shown by the Notch signaling modification. Therefore, we believe that comparing the different phenotypes in 3D culture models originating from the same CSCs would provide a more thorough understanding of the effects of the tumour microenvironment on CSC characteristics.

## Methods

### Cell lines

An LGR5-positive colon cancer stem cell line PLR123 was cultured in cell culture flasks (Corning) in SCM which consisted of DMEM/F-12 (Cat# 11330-057) supplemented with 1× Antibiotic-Antimycotic, 1× N-2 Supplement, 4 mg/mL AlbuMAX I Lipid-Rich BSA, 20 ng/mL human EGF, 20 μg/mL human insulin (all from Thermo Fisher Scientific), 2.9 mg/mL glucose (Merck, total 6.0 mg/mL glucose), 4 μg/mL heparin (Merck), and 10 ng/mL human FGF2 (Reprocell) at 37 °C under 5% CO_2_ as described previously^8^. The colorectal cancer cell lines LoVo and LS174T (both from ATCC) were grown in F-12K (Thermo Fisher Scientific) supplemented with 10% heat-inactivated fetal bovine serum (FBS) or Eagle’s MEM supplemented with 1% Non-Essential Amino Acids Solution, 1 mM Sodium Pyruvate (all from Thermo Fisher Scientific), and 10% FBS, respectively, at 37 °C under 5% CO_2_. The cell lines were adapted to SCM before used in 3D cultures.

### Animal experiments

PLR123 cells were dissociated with Accutase (Nacalai Tesque), suspended in SCM with 50% Matrigel (Corning, Cat# 354234), and 100 cells/30 μL were surgically implanted into the cecum of a NOG mouse (Central Institute for Experimental Animals) under isofluorane anesthesia. Alternatively, PLR123 cells were suspended in PBS and 5 × 10^5^ cells/200 μL were injected intravenously to the tail vein of a NOG mouse. All studies involving animal subjects were performed in accordance with relevant guidelines and regulations approved by the Animal Care and Use Committee at PharmaLogicals Research Pte. Ltd. and the Ethical Committee for Treatment of Laboratory Animals at Chugai Pharmaceutical Co., Ltd.

### 3D cultures

Cells were suspended in SCM with 50% Matrigel (growth factor reduced, Corning, Cat# 356231) and 150 cells/20 μL/well was seeded into standard or clear-bottomed 96-well culture plates (μClear Plates, Greiner or CellCarrier-96 Ultra, PerkinElmer), centrifuged at 1200 rpm for 3 min at 4 °C, and polymerised at 37 °C. The cell number was reduced to 75 cells/20 μL/well for whole mount immunostaining when 3D cultures were 7 and 10 days old. The wells were pre-coated with 20–30 μL of 50% Matrigel solution. Finally, 200 μL of SCM supplemented with 1 mM NAC (Merck) was applied and the plates were incubated at 37 °C under 5% CO_2_ for stemCO culture. For diffCO culture, ΔEFHI medium was used in place of SCM and incubated at a 5% O_2_, 5% CO_2_ atmosphere. The medium was replaced every 2–4 days. 3D culture growth was measured using CellTiter-Glo 3D assay (Promega) after solubilizing Matrigel with dispase (Dispase I, FUJIFILM Wako Pure Chemical). For Notch signal modification, 20 μM JAG-1 peptide (AnaSpec), 10 μM DAPT (Selleck Chemicals), or DMSO control was added to the culture on Day 3, and the medium was replaced every 2–3 days. In experiments for switching culture conditions or long-term 3D cultures, stemCO or diffCO were collected by pipetting, treated with dispase, and fragmented by syringing using a 25- or 27-gauge needle for passage. Bright-field images were obtained with an inverted microscope (IX83, Olympus).

### Histological examination

The lung and liver of NOG mice engrafted with PLR123 cells were fixed with 4% paraformaldehyde (PFA) at 4 °C for 24 hours and embedded in paraffin by the AMeX method^39^. Thin sections were prepared for immunofluorescence and histopathological examination. In the intravenous injection model, there was sufficient tumour tissue for histopathological analysis in the hematoxylin and eosin (HE)-stained slides for all 5 animals, and for immunofluorescence analysis in 3/5 animals. Histopathologically, HE slides were prepared and read under a light microscope. A detailed examination of the morphological difference between the tumours in the lung and liver was carried out for the intravenous injection model. A differentiation grade was designated by the difference in histopathological features as follows: 1, clusters of cells with no duct formation; 2, clusters of cells with small duct-like structures, with unclear apicobasal polarisation of tumour cells; 3, clear formation of ductal structures, with clear apicobasal polarisation and differentiation to villus enterocytes and goblet cells; 4, ductal structures that mimic intestinal lumen, with villus enterocytes, goblet cells, and also necrosis of tumour cells. The percentage of area for each grade was determined and multiplied with the grade number. Then the product for each grade was added, and the sum was designated as the histology score for each animal. For immunofluorescence staining of LGR5 the tyramide signal amplification method was applied. Briefly, an anti-human LGR5 antibody (Clone 2U2E-2)^8^ was applied as the primary antibody, followed by a labeled polymer reagent (EnVision+ Single Reagents, HRP. Mouse, Agilent) as the secondary antibody, and visualised by Alexa Fluor 488-labeled tyramide (Thermo Fisher Scientific). The slides were also stained with DAPI (Thermo Fisher Scientific). A Nikon A1 + confocal microscope (Nikon) was used to examine the slides, according to the method described by Yamazaki *et al*.^40^. In the intravenous injection model, LGR5 positivity was further evaluated by the following scoring method: Images of a representative area (211 × 253 μm) for tumours in the lung and liver for each case were selected. The total number of tumour cells in each area was determined by counting the number of tumour cell nuclei. The positivity of LGR5 cells was graded as 0, negative; 1, positive (small dotty staining in the cytoplasm of tumour cells); 2, strong positive (large patchy staining in the cytoplasm of tumour cells). The density of cells for each grade was calculated as the ratio to the total number of tumour cells. An LGR5 score was designated as the sum of cell density × LGR5 grade.

For evaluation of 3D cultures, paraffin blocks of stemCO and diffCO were prepared using a method described previously^41^. Briefly the 3D cultures were fixed with 4% PFA at room temperature for 30 min and Matrigel was mechanically dissociated by pipetting. Then the organoids were collected, and embedded in paraffin by the AMeX method. HE slides were prepared for all samples. Additionally, an Alcian blue-periodic acid-Schiff (AB-PAS) reaction method, immunohistochemical staining for Ki-67, and immunofluorescence staining for LGR5 were also carried out. Briefly, for AB-PAS, 1% Alcian blue solution (pH 2.5, Muto Pure Chemicals) and Schiff’s solution (Muto Pure Chemicals) were used. For immunohistochemical staining of Ki-67, a polymer method was carried out as previously described^41^. Briefly, an anti-human Ki-67 antibody (Clone MIB-1, Agilent) was applied as the primary antibody after antigen retrieval in Target Retrieval Solution (Agilent). A labeled polymer reagent (EnVision+ Single Reagents, HRP. Mouse, Agilent) was applied as the secondary antibody. Isotype-matched control antibody was applied as negative control. The slides were counterstained with hematoxylin, read under a light microscope, and whole digital slide images were obtained with a virtual microscopy system (Aperio AT2, Leica Biosystems). The images were analyzed for Ki-67 labeling index using the Halo software (v2.3.2089.34, Indica Labs) and Indica Labs cytonuclear v1.6 algorithm (Indica Labs). The total number of cells evaluated was 1765 for stemCOs, and 9156 for diffCOs. For the immunofluorescence observation of LGR5, the method used for the NOG mouse models was applied.

### Whole mount immunostaining for 3D cultures

After removing the medium from the 3D cultures, 200 μL of PBS containing 4% PFA and 1% Triton X-100 was added and incubated for 1 hr on ice. StemCO and diffCO attached to the bottom of each well were carefully washed with PBS supplemented with 1% bovine serum albumin (1%BSA/PBS), and further incubated with blocking buffer (BlockAid Blocking Solution, Thermo Fisher Scientific). Primary antibodies [Alexa Fluor 555-labeled anti-Ki-67 antibody (Clone B56, BD Biosciences), anti-CK20 antibody (Clone SA35-03, Thermo Fisher Scientific), Alexa Fluor 488-labeled anti-Integrin α6 antibody (Clone GoH3, Biolegend), anti-phospho-Ezrin antibody (Clone 48G2, Cell Signaling Technology), or anti-LGR5 antibody (Clone 2L36)^8^] in blocking buffer were added and incubated for 3–4 days at 4 °C, then washed with 1%BSA/PBS and incubated overnight at 4 °C with secondary antibody [Alexa Fluor 488-labeled anti-rabbit IgG antibody (Thermo Fisher Scientific), Alexa Fluor 555-labeled anti-rabbit IgG antibody (Cell Signaling Technology), or Alexa Fluor 555-labeled anti-mouse IgG2a antibody (Thermo Fisher Scientific)] in blocking buffer. Finally the wells were incubated with Alexa Fluor 647-phalloidin, Phalloidin-DyLight 650, or rhodamine-phalloidin and 1 μg/mL DAPI (all Thermo Fisher Scientific). The wells were then incubated with SeeDB2G Solution 1 [1/3× Omnipaque350 (Daiichi-Sankyo) with 2% saponin (Nacalai Tesque)], Solution 2 (1/2× Omnipaque350 with 2% saponin), and Solution 3 (1× Omnipaque350 with 2% saponin) for 30 min each at room temperature^42^ to clear the 3D cultures, which were observed with a confocal fluorescence microscope (C1 or A1, Nikon). For Day 3 and Day 5 cultures, stemCO and diffCO were collected by pipetting after fixation and solubilisation and immunostaining procedure was carried out in low adsorption tubes. Tissue clearing was omitted.

### Confocal live imaging for 3D cultures

PLR123 cells were transfected with LifeAct-TagRFP2 (Ibidi) by electroporation (Nucleofector, Lonza). After selecting stable transformants using G418 (Thermo Fisher Scientific), RFP-positive cells were sorted by flowcytometer (FACS AriaIII, Becton Dickinson), and the cells were maintained without G418. Day 3 3D culture was set under a Confocal Quantitative Image Cytometer CQ1 (Yokogawa Electric) and bright-field and confocal fluorescent images (excitation 561 nm) were monitored at 8-h intervals until Day 10.

### Transmission electron microscopy for 3D cultures

3D cultures were fixed with 4% PFA at room temperature for 30 min and Matrigel was mechanically dissociated by pipetting. The samples were further fixed with 2% glutaraldehyde and 2% PFA in 0.1 M cacodylate buffer for 2 hours at 4 °C followed by post-fix with 1% osmium tetroxide in 0.1 M cacodylate buffer for 1.5 hours at 4 °C. The samples were then embedded in 1% agar, cut into 1 mm^3^ cubes, dehydrated in a series of ethanol, replaced with propylene oxide, and embedded in epoxy resin (Quetol812, Nisshin EM). Resin was heat polymerised. Ultrathin (60–70 nm) sections were prepared using a ultramicrotome (Leica EM UC7, Leica Microsystems), double-stained with uranyl acetate and lead citrate, and observed with a transmission electron microscope (HT7700, Hitachi High-Technologies).

### Colony formation assay

StemCO and diffCO were dissociated into single cells with TrypLE Select (10×) (Thermo Fisher Scientific) and cell aggregates were removed with a 20 μm cell strainer (pluriSelect Life Science). The cells were then suspended in SCM with 0.4% agar (CytoSelect 96-Well Cell Transformation Assay, Cell Biolabs) and 10,000 cells/well were seeded into 96-well culture plates according to the manufacturer. The wells were applied with 100 μL of SCM and incubated at 37 °C under 5% CO_2_ for 7 days. The cells were counted by CyQuant GR Dye according to the manufacturer.

### Quantitative RT-PCR

Total RNA was extracted from 3D cultures or from PLR123 monolayer cells using Trizol (Thermo Fisher Scientific). Quantitative RT-PCR analysis was performed in duplicate for each gene on StepOnePlus Real-Time PCR System (Thermo Fisher Scientific) using SYBR Green (Thermo Fisher Scientific) with the total RNA as templates. *GAPDH* was used as an internal control. Fold difference in gene expression was determined by the 2-ΔΔCt method. The sequence of the primers for PCR are shown in Supplementary Table S1.

### Western blots

Matrigel was solubilised by Cell Recovery Solution (Corning) and stemCO and diffCO were lysed with RIPA Buffer (FUJIFILM Wako Pure Chemical). Lysates were processed following standard procedures. Primary antibodies against cleaved Notch1 (Clone D3B8, Cell Signaling Technology) and β–actin (Clone AC-15, Merck) were used.

### Statistical analysis

Statistical analyses were performed using the JMP version 11.2.1 software (SAS Institute). Statistical significance was determined by the Student t test between stemCO and diffCO in gene expression analysis and colony formation assay. The Dunnett’s multiple comparison test (two-tailed) and the Chi-squared test were employed against the DMSO control group in the analysis of the size of stemCO and diffCO and the ratio of the lumen formation, respectively. A p-value of <0.05 was considered statistically significant.

## Supplementary information

Supplementary Information.

## Data Availability

All data generated or analysed during this study are included in this published article.
