# Supplementary material for: Three-dimensional culture models mimic colon cancer heterogeneity induced by different microenvironments
Source: Sci Rep. 2020 Feb 21;10:3156. doi: 10.1038/s41598-020-60145-9 (PMC7035265; doi:10.1038/s41598-020-60145-9)
Supplement: Supplementary file 1 — Supplementary Information. [file 41598_2020_60145_MOESM1_ESM.pdf]

Three-dimensional culture models mimic colon cancer heterogeneity induced by different microenvironments

Shigeto Kawai[1*], Masaki Yamazaki[2], Keita Shibuya[1], Masaya Yamazaki[1], Etsuko Fujii[1, 2], Kiyotaka Nakano[1] & Masami Suzuki[1, 2]

Affiliation:

[1]Department for Research Division 1, Forerunner Pharma Research Co., Ltd., 4–6–1, Komaba, Meguro-ku, Tokyo 153–8904, Japan

[2]Research Division, Chugai Pharmaceutical Co., Ltd., 1–135, Komakado, Gotemba, Shizuoka 412–8513, Japan

[*]kawaisgt@chugai-pharm.co.jp

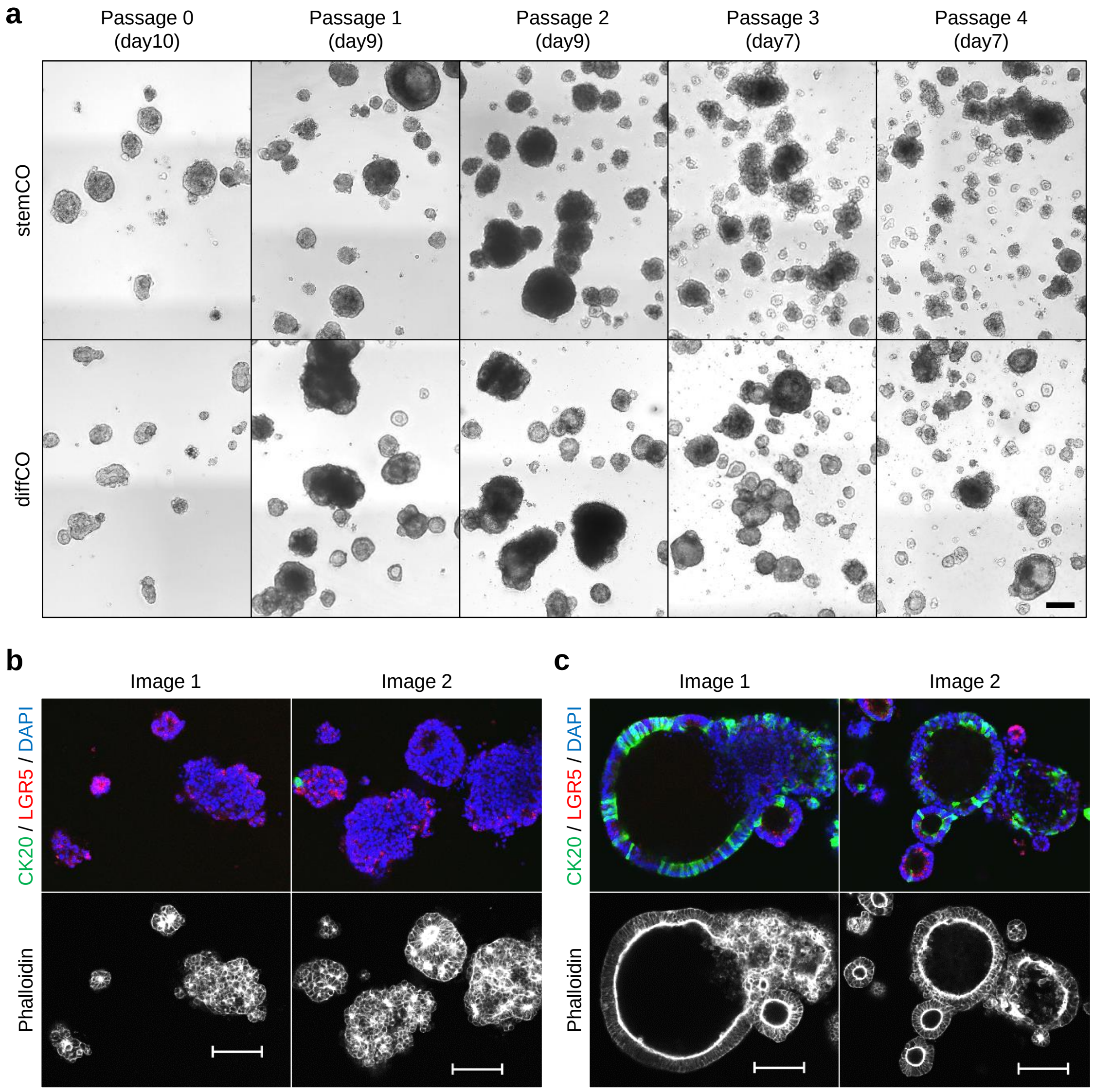

Supplemental Figure S1. Long term culture of stemCO and diffCO. (a) stemCO and diffCO were serially passaged in each culture conditions by fragmentation as described in Methods. Bright-field images at each passage are shown. Number of passages and culture periods for each are indicated in the figure. stemCO (b) and diffCO (c) cultured for total 42 days were stained with anti-CK20 antibody (green), anti-LGR5 antibody (red), phalloidin (white), and DAPI (blue) and observed by confocal microscopy. Scale bars = 200 µm (a) or 100 µm (b,c).

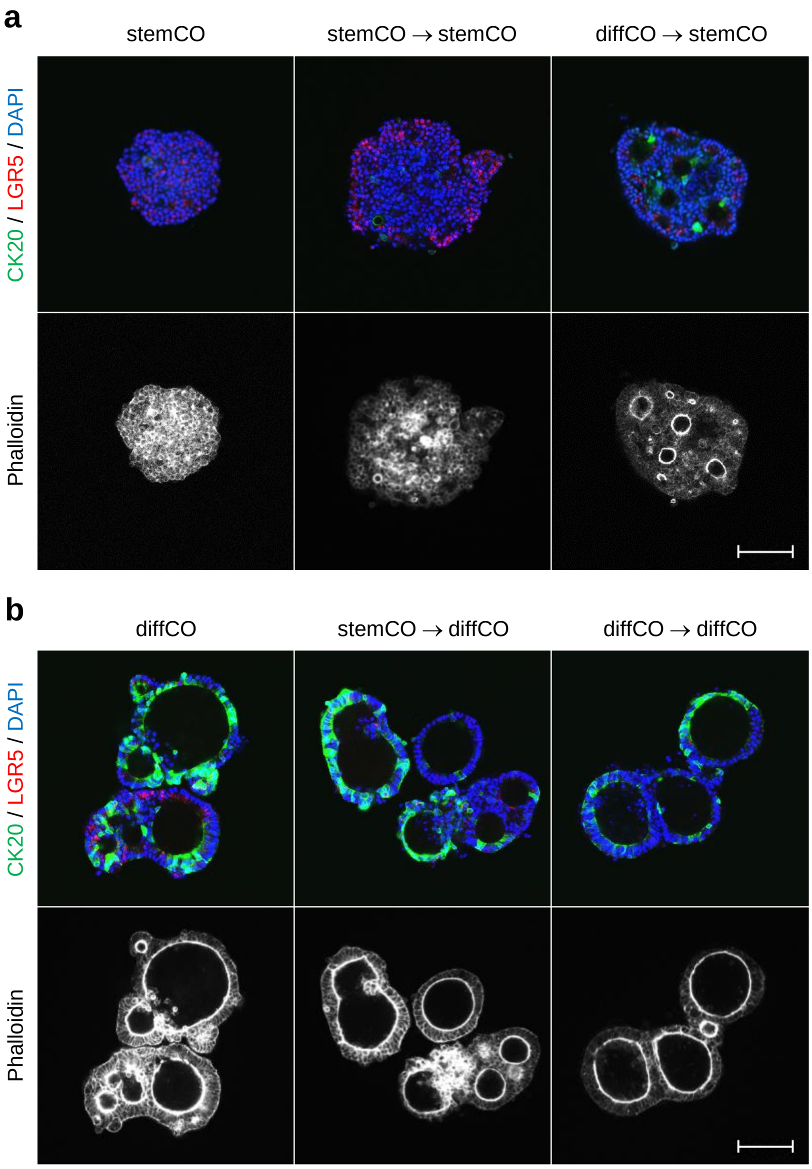

Supplemental Figure S2. Switching stemCO and diffCO culture conditions. Day 10 stemCO and diffCO were fragmented as described in Methods and cultured in stemCO (a) or diffCO culture conditions (b) for a further 10 days. They were stained with anti-CK20 antibody (green), anti-LGR5 antibody (red), phalloidin (white), and DAPI (blue) and observed by confocal microscopy. Day 10 stemCO (a) or diffCO (b) served as controls. Scale bars = 100 μm.

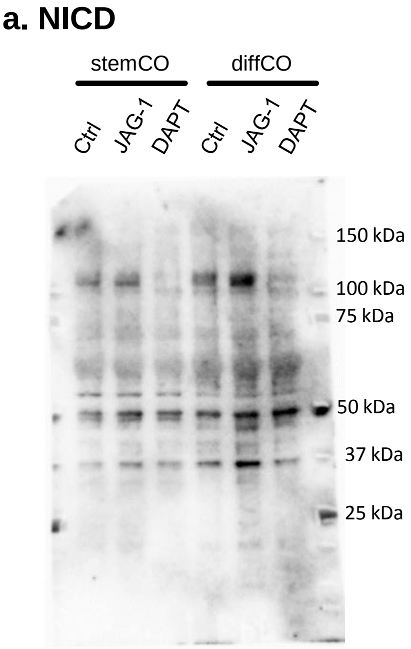

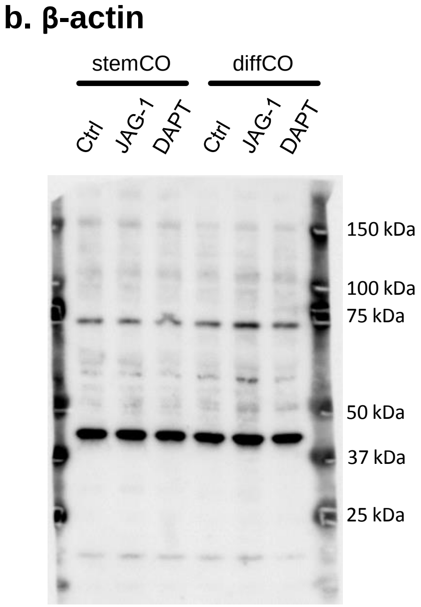

Supplemental Figure S3. Whole images of Western blot analysis.

Supplemental Table S1. Primers used for quantitative RT-PCR analysis

Human gene	Forw ard primer	Reverse primer
LGR5	AATCCCCTGCCCAGTCTC	CCCTTGGGAATGTATGTCAGA
CD44	AGCAACCAAGAGGCAAGAAA	GTGTGGTTGAAATGGTGCTG
PROM1	GGAAACTAAGAAGTATGGGAGAACA	CGATGCCACTTTCTCACTGAT
CDX1	CAAGTACCGCGTGGTCTACA	CCAGATCTTCACCTGCCGTT
CDX2	ATCACCATCCGGAGGAAAG	TGCGGTTCTGAAACCAGATT
KRT20	TGTCCTGCAAATTGATAATGCT	AGACGTATTCCTCTCTCAGTCTCATA
HES1	GAAGCACCTCCGGAACCT	GTCACCTCGTTCATGCACTC
NRARP	GCGTTGTGAAGGCAACAGAG	GGGAGGCTAAAAAGGGGCAA
GAPDH	GCACCGTCAAGGCTGAGAACG	TGGTGAAGACGCCAGTGGACT
